# Pancreaticoduodenectomy in a patient with intestinal malrotation and distal cholangiocarcinoma: a case report and review of the literature

**DOI:** 10.1186/s13256-020-02468-9

**Published:** 2020-09-16

**Authors:** Nassir Alhaboob Arabi, Areej Abdalla Abdoun, Mohamed Osama Ali, Saria Kabashi Elhaj, Sawsan Abuelgassim Mohd.

**Affiliations:** Department of Gastrointestinal and Hepato-Pancreatico-Biliary Surgery, Ibn Sina Specialized Hospital, Khartoum, Sudan

**Keywords:** Malrotation, Nonrotation, Midgut anomaly, Pancreaticoduodenectomy, Vascular anomaly

## Abstract

**Background:**

A combination of intestinal malrotation and distal cholangiocarcinoma is considered a rare condition and poses some difficulties in surgical management. We present a case of a patient with asymptomatic nonrotation of the midgut with a concomitant distal cholangiocarcinoma who underwent successful pancreaticoduodenectomy.

**Case presentation:**

A 52-year-old Sudanese man presented to our hospital with progressive painless jaundice associated with dark urine, pale stool, and itching for the last 2 months. He had no other complaint or significant previous medical history apart from being an ex-smoker. His clinical examination revealed a palpable gallbladder and scratch mark. His other systems were unremarkable. His blood test results showed a normal complete blood count, elevated total bilirubin (mainly direct bilirubin), elevated alkaline phosphatase, and normal cancer antigen 19-9 and carcinoembryonic antigen. Ultrasound, computed tomography of the abdomen, and magnetic resonance cholangiopancreatography showed a dilated intrahepatic and extrahepatic biliary system down to the distal part, where the lumen was obstructed by a periampullary mass measuring 2.4 by 2.1 cm. The patient’s gallbladder was distended. He had no liver metastases or ascites and few lymph nodes. Inversion of the superior mesenteric artery and superior mesenteric vein but no invasion was seen, and malrotation of the bowel was observed with the large bowel on the left side and the small bowel to the right of the abdomen. Endoscopic retrograde cholangiopancreatography showed a fleshy ampulla that was stented. Laparotomy showed malrotation, with the duodenum straight on the right side of the midline, and Ladd’s band crossed the second portion of the duodenum. The vessels were approached from the lateral side meticulously after kocherization of the duodenum and pancreas, dissection along an extended portion of the superior mesenteric artery to assure preservation of the superior mesenteric artery and branches going to the jejunum, Ladd’s procedure, division of the jejunum 10 cm below the uncinate process of pancreas, and modified pancreaticoduodenectomy were performed, and anastomoses were performed in the standard fashion. The patient had an uneventful postoperative course, started oral feeding after 5 days, and discharged to home on day 10 for regular follow-up. Histopathology confirmed distal cholangiocarcinoma, and the patient was referred for further oncological management.

**Conclusions:**

Pancreaticoduodenectomy can be safely performed in patients with intestinal malrotation with some modifications of the standard approach. Meticulous dissection after preoperative identification of vascular anomaly and a lateral approach are of great help to reduce morbidity.

## Background

Intestinal malrotation occurs during the sixth to tenth weeks of embryological development. It affects the position and peritoneal attachments of the bowel with alteration in vascular and anatomic relationships [1, 2], and it has three stages: nonrotation (absent), incomplete rotation, and malfixation [2, 3]. Cholangiocarcinoma (CCA) is a heterogeneous group of tumors derived from cells of the biliary tree that represent the second most frequent primary liver tumor [4].

A combination of intestinal malrotation and distal CCA is considered a rare condition and is challenging, especially with regard to surgical management. Although malrotation is conventionally viewed as a pediatric condition, it can be found in adult patients in nearly 10–20% of cases, and despite this congenital development, it rarely presents with gastrointestinal neoplasms [5, 6].

## Case presentation

**Fig. 1:**
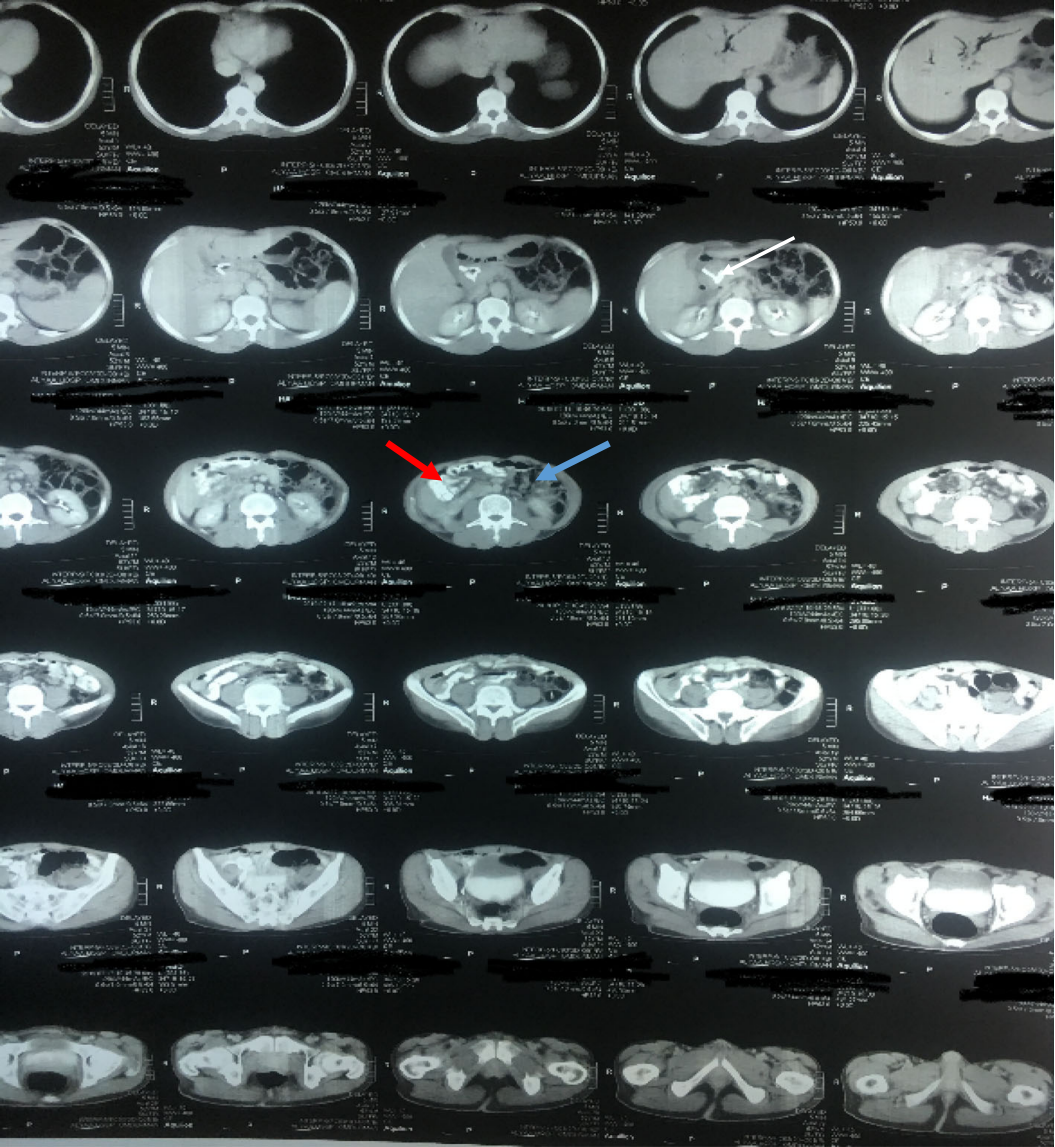
Computed tomographic scan of the abdomen showing malrotation of the bowel, periampullary mass, dilated common bile duct, and stent inside (white arrow), small bowel on the Right side (red arrow) and large bowel on the left (blue arrow)

**Fig. 2:**
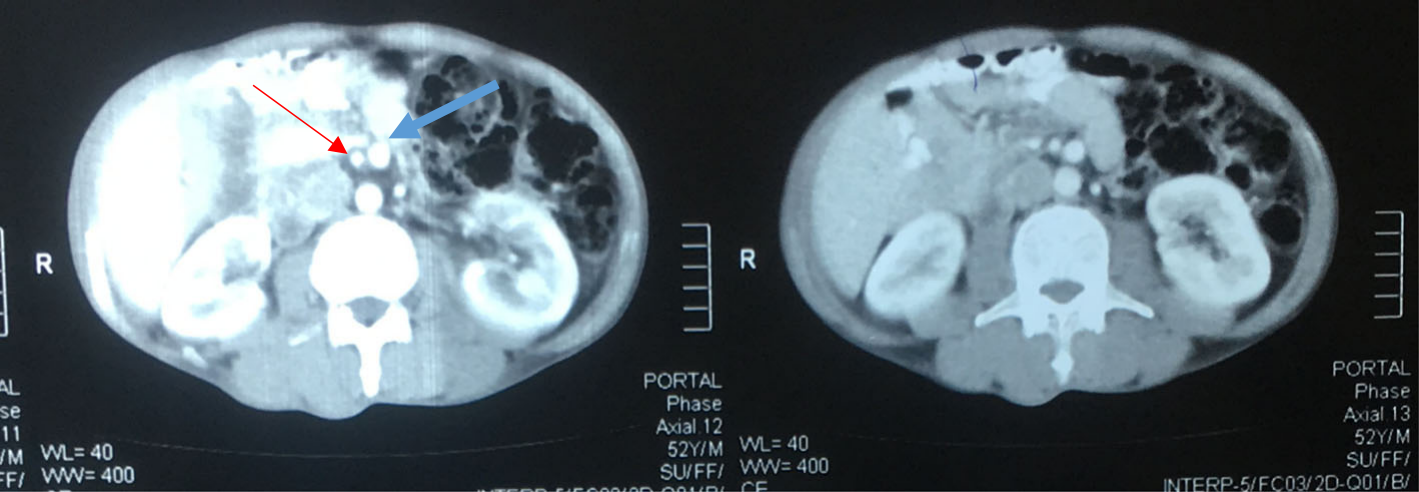
Computed tomographic scan of the abdomen showing inversion of superior mesenteric artery (red arrow) and superior mesenteric vein (blue arrow)

**Fig. 3:**
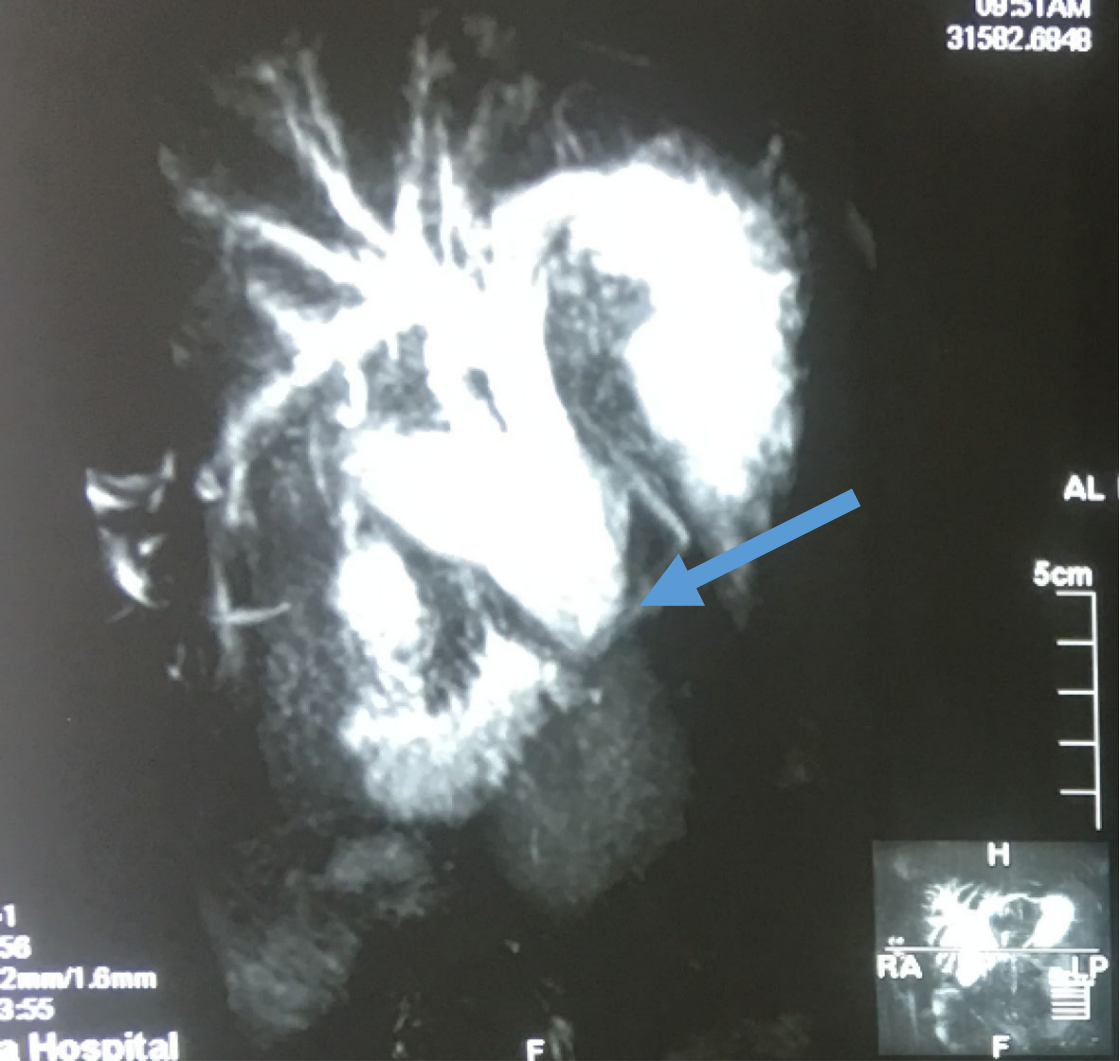
Magnetic resonance cholangiopancreatography showing dilated intrahepatic and extrahepatic biliary system down to the distal part (arrow)

**Fig. 4:**
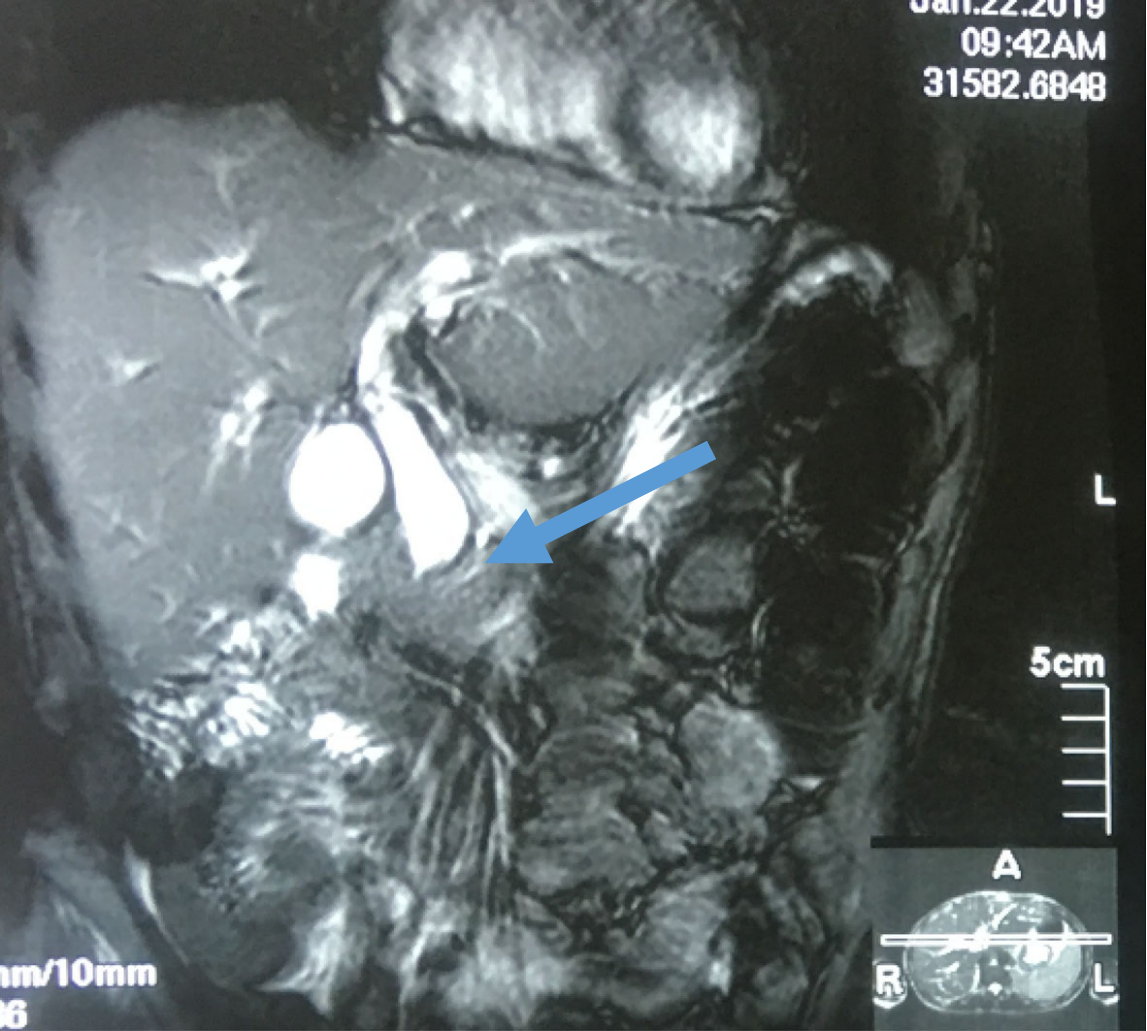
Magnetic resonance cholangiopancreatography showing dilated intrahepatic and extrahepatic biliary system down to the distal part, where it is obstructed by a mass (arrow)

A 52-year-old Sudanese man presented to our outpatient clinic with progressive painless jaundice associated with dark urine, pale stool, and itching for the last 2 months. He had no fever or alteration in the level of consciousness. He had no other complaint and no significant previous medical history apart from being an ex-smoker. His clinical examination revealed deep jaundice and a palpable gallbladder and scratch mark. His other systems were unremarkable. The results of his blood test showed a normal complete blood count, elevated total bilirubin (mainly direct bilirubin), and elevated alkaline phosphatase but normal aspartate transaminase and alanine transaminase, normal cancer antigen 19-9 and carcinoembryonic antigen. Ultrasound, computed tomography of the abdomen (Figs. 1 and 2), and magnetic resonance cholangiopancreatography (Figs. 3 and 4) showed a dilated intrahepatic and extrahepatic biliary system down to the distal part, where the lumen was obstructed by a periampullary mass measuring 2.4 by 2.1 cm. The patient’s gallbladder was distended, but he had no liver metastases or ascites. Few lymph nodes seen. We observed inversion of the superior mesenteric artery (SMA) and superior mesenteric vein (SMV) but no tumor invasion. We also observed malrotation of the bowel with the large bowel on the left side and the small bowel to the right side of the abdomen. Endoscopic retrograde cholangiopancreatography showed a fleshy ampulla and a dilated extrahepatic and intrahepatic biliary system with a distal malignant-looking mass, and it was stented.

**Fig. 5:**
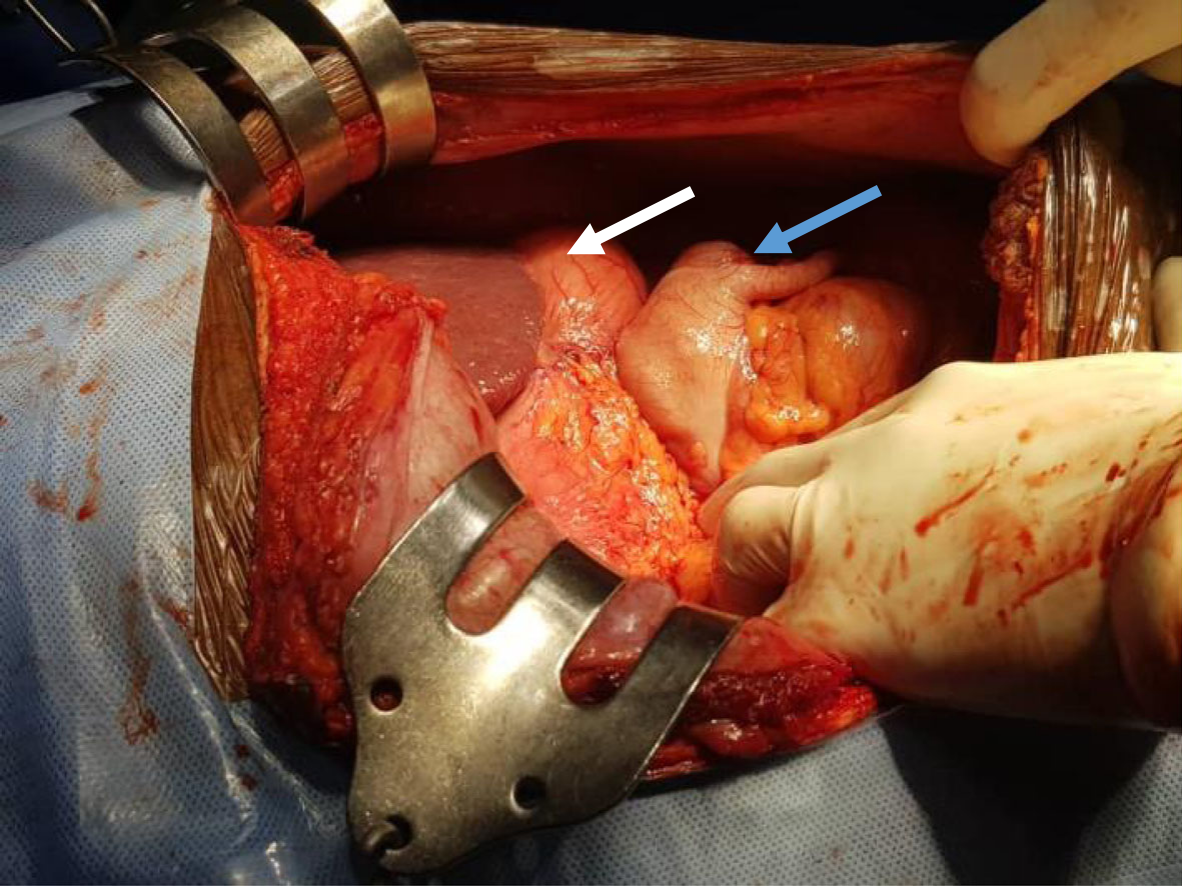
Intraoperative picture showing the cecum and appendix on the left hypochondrium (blue arrow) just below the stomach (white arrow)

**Fig. 6:**
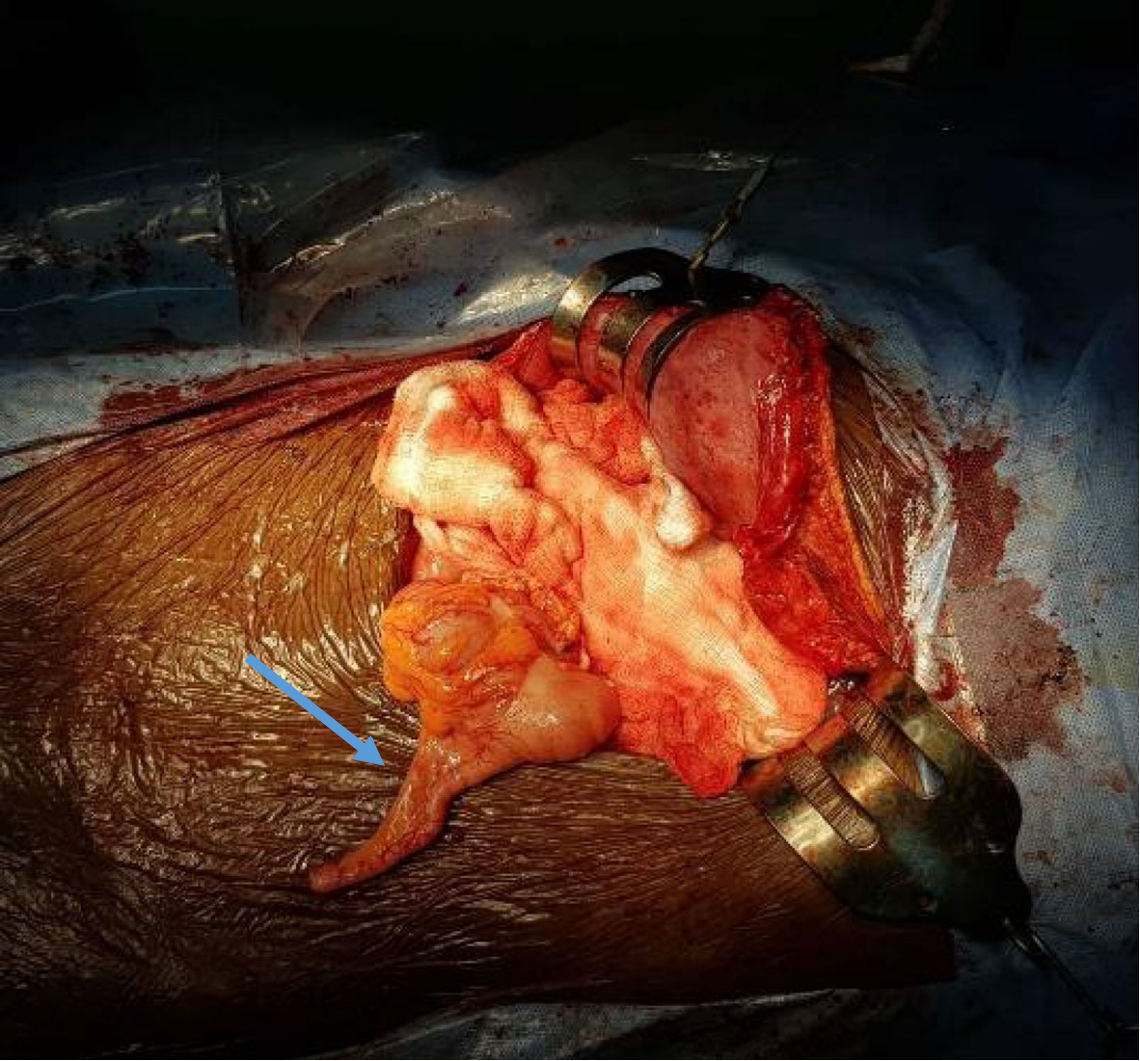
Intraoperative picture showing the cecum and appendix (arrow) on the left hypochondrium

**Fig. 7:**
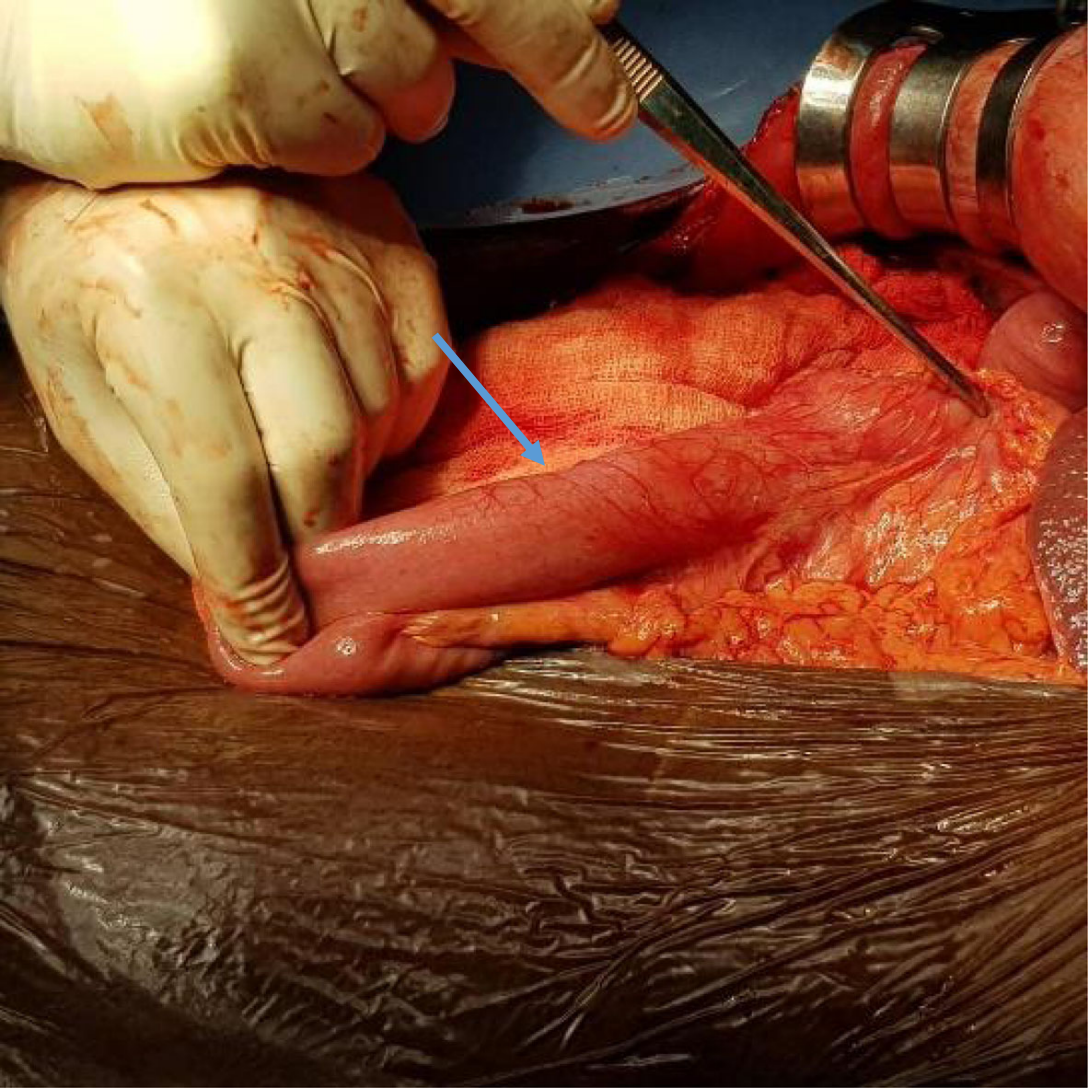
Intraoperative picture showing failure of the duodenum to cross the midline (straight duodenum) (arrow the 3rd part of the duodenum)

Based on the above data, a decision was made to perform laparotomy and pancreaticoduodenectomy (PD). Laparotomy showed that the small bowel was on the right side and the large bowel was on the left side of the midline, and the cecum and appendix were seen on the left upper quadrant (Figs. 5 and 6). There was no duodenojejunal flexure, and the duodenum was straight on the right side of the midline (Fig. 7). The superior mesenteric artery and vein were altered in position. Ladd’s bands were located across the supposed second portion of the duodenum. A periampullary palpable mass was detected, nearly 3 cm in size, resectable and separable from the vessels. Few small lymph nodes and no ascites, liver metastases, or omental implant were seen. The decision to proceed with PD was made. Difficulty was encountered due to the vascular anomaly because the SMA was on the right side of the SMV. We performed meticulous dissection, hoping to avoid deformed anatomy and unexpected bleeding. Initially, Ladd’s band was divided, followed by kocherization of the duodenum and the head of the pancreas. The vessels were approached from the lateral side, and dissection of the uncinate process was performed with caution in order to avoid inadvertent injury. Then dissection along an extended portion of the superior mesenteric artery was required to assure preservation of the superior mesenteric artery and branches going to the jejunum, and distal division of the jejunum was performed 10 cm beyond the uncinate process of the pancreas because there was no ligament of Treitz to serve as a landmark for the distal resection margin. Subsequently, creation of a retropancreatic tunnel, division of the duodenum distal to the pylorus, division of the gastroduodenal artery, cholecystectomy with the distal common bile duct (CBD), and division of the pancreas en bloc were carried out according to standard PD. Pancreaticojejunal, hepaticojejunal, and gastrojejunal anastomoses were performed in the standard fashion. Additionally, appendectomy was performed (Ladd’s procedure). The patient had an uneventful postoperative course, started oral feeding after 5 days, and discharged to home on day 10 for regular follow-up. Histopathology results confirmed distal CCA, and the patient was referred for further oncological management.

## Discussion and conclusions

Malrotation is a congenital intestinal gestational disorder that affects not only the positioning of the midgut but also its vascular supply, and sometimes the ligament of Treitz is in abnormal position or even absent. It is generally a disease of childhood, but it can have significant implications later in adult surgeries as well, and sometimes it can cause recurrent abdominal pain and volvulus [6–8], but it rarely presents with a gastrointestinal neoplasm [5].

CCA is a rare malignancy and accounts for 2% of all malignancies. It arises from the malignant growth of the epithelial lining of the bile ducts and can be found all along the biliary tree. It can be classified into subtypes based on location: intrahepatic, perihilar, and distal [9], with the latter considered among periampullary tumors.

CCAs are usually identified at advanced stages [4], and malignancies in the extrahepatic bile duct exhibit similar metastatic patterns [10]. A main challenge in diagnosing CCA is the fact that it is difficult to obtain tumor tissue for histological examination. Thus, a major clinical obstacle is the establishment of the correct diagnosis, and imaging plays an important role in the detection and characterization of CCA with or without guiding biopsy procedures and allowing staging of the tumor [5, 11].

PD for periampullary tumors remains the standard procedure and can be performed safely with low mortality and morbidity rates [12]. During PD, arterial anomalies can increase the operative complexity of surgery, which is determined by the course of these arteries, but they do not usually compromise the safety of the procedure or its oncological outcome [13]. Body mass index, neutrophil-to-lymphocyte ratio, margin status, locoregional lymph node metastases, and the use of chemotherapy are the most important determinants of postsurgical outcomes [9, 14, 15].

To the best of our knowledge, few case reports in the literature mention patients with intestinal malrotation requiring PD. Plackett *et al.* reported a case of a 69-year-old woman with a 2-cm pancreatic head mass and nonrotation of the intestines in whom an exploratory laparotomy was performed that confirmed the presence of intestinal nonrotation with the small bowel in the right abdomen, absence of a ligament of Treitz, and large bowel all within the left abdomen. Ladd’s bands were present across the second and third portions of the duodenum and were divided [6]. Additionally, Mateo *et al.* [1] reported cases of three adult patients with congenital rotational disorders requiring PD. Two of them had biliopancreatic tumors, and one cadaveric donor underwent total pancreatectomy during pancreas allograft procurement. All patients had arterial and venous anomalies around the celiac trunk and mesenteric vessels, respectively. The midgut and hindgut in each case were shifted toward opposite sides of the abdominal cavity. Modifications of the standard approach to PD were made, and outcomes were favorable in each case. Each patient showed anatomic abnormalities with the need for identifying vascular structures through their expected course and location before parenchymal division or ligation of any vessel. This approach becomes crucial in cases of vascular anomalies, such as ones occurring in congenital malformations, and can be used in similar situations encountered during PD [1]. Baer *et al.* [16] studied five patients who presented with resectable periampullary neoplasms (two pancreatic adenocarcinoma, one ampullary carcinoma, one duodenal adenocarcinoma, and one intraductal papillary mucinous neoplasm). Preoperative imaging showed that they had intestinal malrotation with inversion of the SMA and SMV and failure of the duodenum to cross the midline. Successful PD was performed in all cases with a modified approach. Aberrant vascular and anatomic locations mandated careful parenchymal resection. Although the SMA was more accessible at the head of the pancreas as it passed to the right of the SMV, the location of intestinal and pancreatic branches required precise identification of their actual as opposed to expected course. Particular attention was paid to the dissection of the uncinate process in order to avoid inadvertent injury to arteries supplying the small bowel. The reconstruction was performed in standard fashion in all cases. All patients tolerated their procedures well. Postoperative complications included one peripancreatic abscess requiring drainage [16].

We conclude that PD can be performed safely in patients with intestinal malrotation, and preoperative understanding of the vascular anatomy, particular attention intraoperatively, and a lateral approach in the dissection will reduce complications. Modifications to the standard approach to PD were needed in our patient.

## Data Availability

The datasets used and/or analyzed during the current study are available from the corresponding author on reasonable request. All medical data, supporting materials, and images are available upon request.

## Abbreviations

CBD: Common bile duct
CCA: Cholangiocarcinoma
PD: Pancreaticoduodenectomy
SMA: Superior mesenteric artery
SMV: Superior mesenteric vein
